# Role of capsaicin-sensitive C-fiber afferents in neuropathic pain-induced synaptic potentiation in the nociceptive amygdala

**DOI:** 10.1186/1744-8069-8-51

**Published:** 2012-07-09

**Authors:** Ayano Nakao, Yukari Takahashi, Masashi Nagase, Ryo Ikeda, Fusao Kato

**Affiliations:** 1 Laboratory of Neurophysiology, Department of Neuroscience, Jikei University School of Medicine, Minato-ku, Tokyo, 105-8461, Japan; 2 Department of Orthopaedics, Jikei University School of Medicine, Minato-ku, Tokyo, 105-8461, Japan; 3 Nagoya University Graduate School of Medicine, Department of Cell Pharmacology, Graduate School of Medicine, Nagoya University, 65 Tsurumai, Showa, Nagoya, Aichi, 466-8550, Japan

**Keywords:** Excitatory postsynaptic currents, Parabrachial nucleus, Tactile allodynia, Emotion, Minimal stimulation, Capsular part of the central nucleus of the amygdala

## Abstract

**Background:**

Neurons in the capsular part of the central nucleus of the amygdala (CeC), a region also called "nociceptive amygdala," receive nociceptive information from the dorsal horn via afferent pathways relayed from the lateral parabrachial nucleus (LPB). As the central amygdala is known to be involved in the acquisition and expression of emotion, this pathway is thought to play central roles in the generation of affective responses to nociceptive inputs. Excitatory synaptic transmission between afferents arising from the LPB and these CeC neurons is potentiated in arthritic, visceral, neuropathic, inflammatory and muscle pain models. In neuropathic pain models following spinal nerve ligation (SNL), in which we previously showed a robust LPB-CeC potentiation, the principal behavioral symptom is tactile allodynia triggered by non-C-fiber low-threshold mechanoreceptor afferents. Conversely, recent anatomical studies have revealed that most of the spinal neurons projecting to the LPB receive C-fiber afferent inputs. Here, we examined the hypothesis that these C-fiber-mediated inputs are necessary for the full establishment of robust synaptic potentiation of LPB-CeC transmission in the rats with neuropathic pain.

**Results:**

Postnatal capsaicin treatment, which has been shown to denervate the C-fibers expressing transient receptor potential vanilloid type-1 (TRPV1) channels, completely abolished eye-wiping responses to capsaicin eye instillation in rats, but this treatment did not affect mechanical allodynia in the nerve-ligated animals. However, the postnatal capsaicin treatment prevented LPB-CeC synaptic potentiation after SNL, unlike in the vehicle-treated rats, primarily due to the decreased incidence of potentiated transmission by elimination of TRPV1-expressing C-fiber afferents.

**Conclusions:**

C-fiber-mediated afferents in the nerve-ligated animals may be a required facilitator of the establishment of nerve injury-evoked synaptic potentiation in the CeC. These inputs might play essential roles in the chronic pain-induced plastic changes in the central network linking nociception and negative emotion.

## Background

Pain has sensory and emotional dimensions. Whereas the sensory dimension stems from somatotopical analysis of the origin and intensity of the nociception, the emotional dimension defines the aversiveness of the nociception, urging individuals to avoid life-threatening situations at various time-scales. This bi-dimensional nature is not necessarily unique to pain because such "emotional" aspects are rather common in various types of primordial sensations such as olfaction [1] and taste [2]. Such emotional aspects of sensation seem to have played significant roles in evolution by increasing survival chances, thus providing a biological and evolutional basis for emotion [3]. Interestingly, in these sensory systems, the aversive information linked with negative emotion is mediated by specific classes of primary receptor systems. For example, neurons in the dorsal domain of the olfactory bulb are involved in detecting innately aversive odors, such as those from predators [1]. Likewise, bitter taste cells are involved in detection of potentially harmful ingredients, such as toxic plant alkaloids in food [4]. However, in contrast to these senses, it remains undetermined for the pain system whether a specific type of nociceptor plays a specific role in establishing the strong link between nociception and emotion in a similar manner to the olfactory or gustatory systems.

In general, nociception is mediated by two classes of afferent fibers: Aδ- and C-fibers. Of these, the following lines of evidence suggest that the information carried by C-fibers is more related to the generation of negative emotion. First, most of the neurons with ascending projection in the spinal lamina I project to the lateral parabrachial nucleus (LPB) and preferentially receive peptidergic C-fiber inputs [5,6]. Second, the LPB then sends excitatory projections to the capsular part of the central amygdala (CeC) [7-10], in which a large majority of the neurons show excitatory responses to noxious stimulation [11]. Third, the central amygdala is one of the important regions that plays essential roles in the expression of aversive signal-induced emotional behaviors, well described as fear conditioned responses [12]. In accordance with these observations, the semi-acute inflammatory pain models, such as the kaolin/carrageenan-induced arthritis, zymosan-induced colitis and acid-induced muscle pain, exhibit robust potentiation of LPB to CeC synaptic transmission (LPB-CeC potentiation) [13,14]. This argues in favor of the involvement of C-fiber afferents activated by inflammation in the establishment of LPB-CeC potentiation. However, in contrast to these inflammatory pain models, such LPB-CeC potentiation is also reported in a hemilateral spinal nerve ligation (SNL) neuropathic pain model [15], in which the tactile allodynia evoked by non-noxious touch is the prominent and predominant chronic pain symptom [16,17]. Such tactile allodynia does not involve C-fiber activation, but rather, is triggered by activation of low-threshold mechanoreceptors and Aβ afferents, which normally do not mediate nociceptor information. Therefore, taking advantage of this afferent fiber specificity of the SNL model, we sought to determine the role of C-fiber primary afferents in the establishment of LPB-CeC synaptic potentiation.

For this purpose, we examined whether experimental denervation of C-fibers expressing TRPV1 channels affects the potentiation of LPB-CeC transmission following SNL. We performed SNL in young rats that underwent neonatal capsaicin treatment in which most of the peripheral fibers expressing TRPV1 channels were irreversibly ablated [18-21], and evaluated LPB-CeC synaptic potentiation and mechanical allodynia. The results indicate that LPB-CeC potentiation in response to spinal nerve injury was prevented by the neonatal pharmacological denervation of TRPV1-expressing fibers, despite the clear manifestation of mechanical allodynia. This suggests that C-fiber-mediated nociceptive inputs are central in establishing the potentiated link between nociception and amygdala network and would influence chronic pain-induced emotional complication [13,22].

## Results

### Reduced capsaicin-induced eye wiping in capsaicin-treated rats

**Figure 1:**
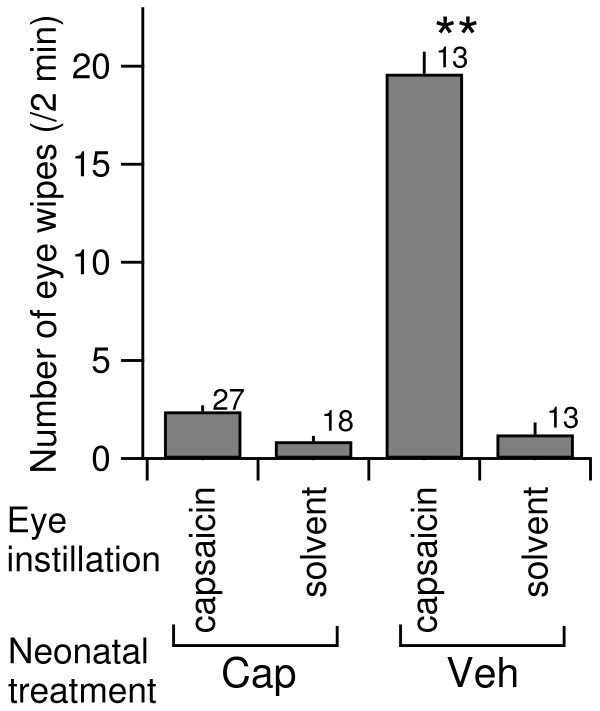
**Effect of neonatal capsaicin treatment on capsaicin-evoked responses at postnatal days 19–20.** Number of eye wipes within 2 min after eye instillation (i.s.) of 0.01% capsaicin or solvent into either eye. Mean ± SEM. Cap and Veh responses measured in 27 capsaicin-treated and 13 vehicle-treated rats, respectively. **, P < 0.01; vs. capsaicin (i.s.) in capsaicin-treated rats and vs. solvent (i.s.) in both capsaicin- and vehicle-treated rats (one-way ANOVA followed by Bonferroni post-hoc correction). The numbers on the top-right shoulder of the bars indicate the number of rats.

Twenty-seven rats were treated with capsaicin 12–48 hours after birth. Thirteen rats were injected with vehicle. On postnatal day 19–20, the eye-wiping behavior in response to the eye instillation of capsaicin was evaluated. The number of eye-wipes in response to 0.01% capsaicin was markedly and significantly reduced in the capsaicin-treated group (the rats with postnatal capsaicin injection) compared with the vehicle-treated group (those with postnatal vehicle injection; Figure 1). While the rats in the vehicle-treated group showed 13–25 eye wipes following capsaicin application (n = 13 rats), those in the capsaicin-treated group showed only 0–6 eye wipes (n = 27). Of these capsaicin-treated rats, 14.8% (n = 4) showed a complete absence of eye wipe responses. Rats in both treatment groups showed only a limited number of eye wipes in response to instillation of the solvent (Figure 1; ranging from 0 to 4 and from 0 to 7 eye wipes in capsaicin- and vehicle-treated groups, respectively). The numbers of eye wipes following solvent instillation were not significantly different between capsaicin-treated and vehicle-treated rats (Figure 1), and, in the capsaicin-treated rats, the numbers of eye wipes following capsaicin and solvent instillation were not significantly different (Figure 1). These results indicate that sensory nerves activated by capsaicin (i.e., those expressing TRPV1 receptors) are almost completely abolished in the capsaicin-treated group [18,23].

### Effect of capsaicin treatment on tactile allodynia

**Figure 2:**
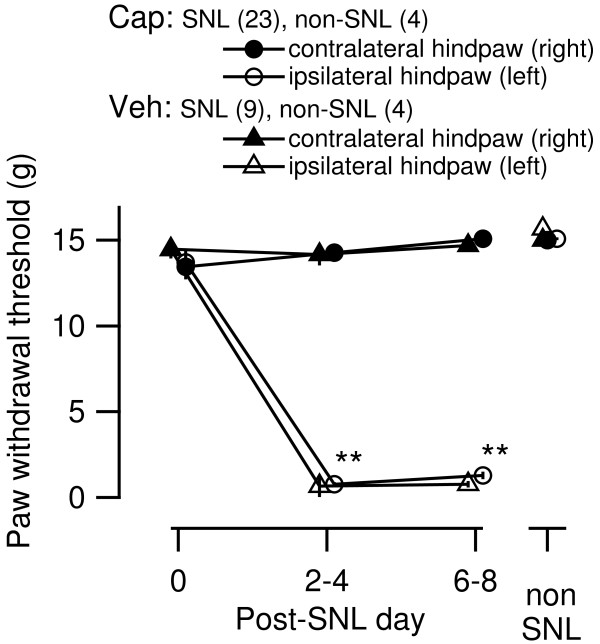
**Effect of neonatal capsaicin treatment on allodynic responses in rats at 7–9 days after unilateral spinal nerve ligation.** Abscissa, the time point at which the measurement was made (day after operation); ordinate, 50%-paw withdrawal threshold estimated according to the responses to von Frey filament stimulation of the right (filled markers) and left (open markers) hindpaw. Nerve ligation was performed on the left spinal nerve for 32 rats. Circles represent the data from capsaicin-treated rats, and triangles indicate the values from the vehicle-treated group. Mean ± SEM. **, P < 0.01 in all comparisons vs. pre-operation values, values for the right hindpaw of both capsaicin- and vehicle-treated rats and values for both right and left hindpaw of the non-nerve ligated (non SNL) rats (one-way ANOVA followed by Bonferroni post-hoc correction). The numbers in parentheses indicate the number of rats.

The SNL was made in 23 capsaicin-treated and 9 vehicle-treated rats. In addition, 4 rats from capsaicin-treated group and 4 rats from vehicle-treated rats did not undergo SNL operation to evaluate the effects of capsaicin-treatment without SNL ("non-SNL" rats). Tactile allodynia was evaluated in all rats at the following 3 times: immediately before the SNL (postoperative day 0), 2–4 days postoperative and immediately before brain isolation for slice preparation (postoperative day 7–9; i.e., P27-P36). The paw withdrawal threshold (PWT) of the left hindlimb (i.e., ipsilateral to the SNL) was markedly and significantly lower than that measured before SNL and was also lower than that measured in the right hindlimb in both capsaicin-treated (circles in Figure 2) and vehicle-treated (triangles) rats at postoperative days 2–4 and 6–8. There was no significant difference between the capsaicin- and vehicle-treated rats in PWT in either of left or right hindlimb at any time point of measurement. Such lack of a detectable difference between capsaicin- and vehicle-treated rats in the decreased PWT on the SNL side confirms the notion that the injured side-specific establishment of tactile allodynia after the SNL does not require capsaicin-sensitive C-fiber afferents [17,24]. The rats without SNL (4 from capsaicin-treated group and 4 from vehicle-treated group) did not show any decrease in PWT as measured at the same postnatal day as the other rats (Figure 2).

### Effect of capsaicin treatment on the side-specific increase in amplitude of the compound EPSC (cEPSC) evoked by LPB-CeC pathway stimulation

We have already demonstrated that excitatory synaptic transmission at LPB-CeC synapses is potentiated predominantly in the CeC contralateral to the SNL side in the rats with L5 SNL [15]. To examine the effect of neonatal capsaicin treatment on such neuropathic pain-related potentiation, we analyzed the synaptic transmission at the LPB-CeC synapses. The rats were decapitated immediately after the final measurement of PWT to prepare brain slices containing the amygdala, for electrophysiological recordings.

**Figure 3:**
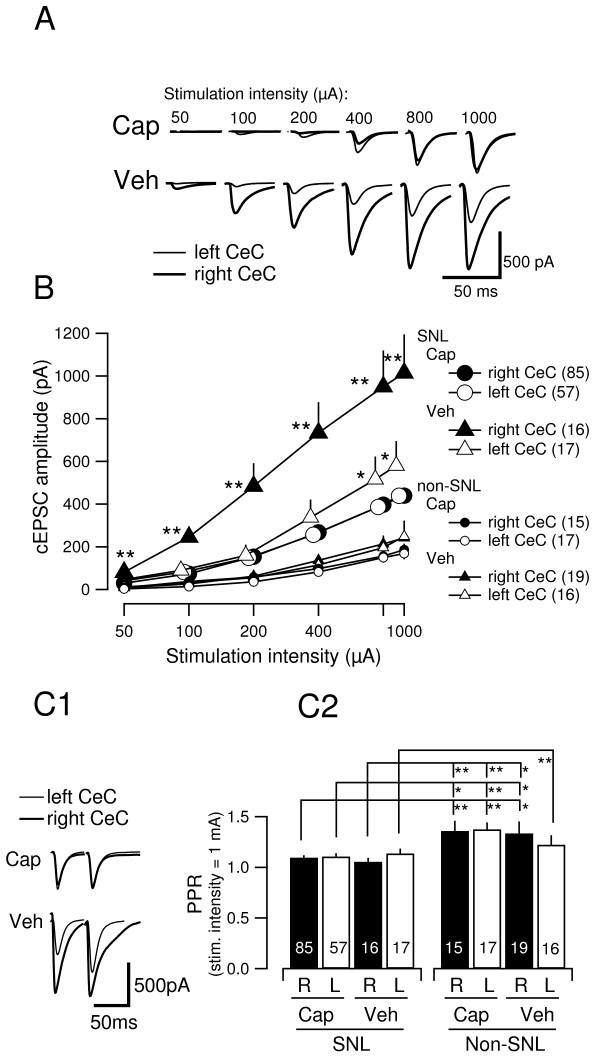
**Effect of capsaicin treatment on cEPSCs evoked by LPB tract stimulation in the CeC in the neuropathic pain model.A**, representative waveforms (averages of 8 to 10 consecutive responses) of cEPSCs evoked by LPB tract stimulation with increasing intensities (from left to right). Top, recordings from a capsaicin-treated rat from left (thin line) and right (thick line) CeC neurons. Bottom, recordings from a vehicle-treated rat. Recordings were from 4 different rats at 7 days after nerve ligation. **B**, input–output relation between the stimulation intensity (abscissa) and cEPSC amplitude (ordinate). Based on the data from rats at 7–9 days after nerve ligation. Large-size markers indicate the values from neurons in the left CeC (open markers) and the right CeC (filled markers) from the capsaicin- (circles) and vehicle- (triangles) treated SNL groups. Small-size markers indicate those values obtained from non-SNL rats. Mean ± SEM. The number of neurons is indicated in the right-side legends for each group. **, P < 0.01; vs. neurons of all other 7 groups (one-way ANOVA followed by Bonferroni post-hoc correction). *, P < 0.05; vs. right CeC in vehicle-treated SNL rat and vs. both sides of CeCs in capsaicin-treated non-SNL rats (one-way ANOVA followed by Bonferroni post-hoc correction). The numbers in parentheses indicate the number of neurons. **C**, Comparison of the paired pulse ratio (PPR) of cEPSCs. 1, representative averaged cEPSC waveforms recorded in bilateral CeC neurons from capsaicin- and vehicle-treated SNL rats. Note that the ratio of the first to the second cEPSC amplitude did not essentially change, despite manifest differences in the amplitude both in vehicle- and capsaicin-treated SNL groups. 2, summary of the results of PPR (as measured for cEPSCs evoked by 1-mA stimulation). Left 4 bars, data from SNL rats; right 4 bars, data from non-SNL rats. There were no significant differences between the capsaicin- and vehicle-treated groups in both SNL and non-SNL groups (Mann–Whitney *U* test). *, P < 0.05; **, P < 0.01 (Mann–Whitney *U* test) between SNL and non-SNL groups. Mean ± SEM. Numbers in the bars indicate the number of neurons.

Figure 3A shows representative traces of compound EPSCs (cEPSCs) evoked by LPB pathway stimulation of increasing intensities in CeC neurons from the capsaicin- (above) and vehicle- (bottom) treated rats. In accordance with our previous report [15], the amplitude of cEPSC in the right CeC (thick traces) was larger than that in the left CeC at each stimulation intensity (from left to right; 50–1000 μA) in vehicle-treated rats (Figure 3A, Veh). In contrast, there was no apparent difference in the cEPSC amplitudes between left and right CeC neurons from a representative capsaicin-treated rat (Figure 3A, Cap). Figure 3B indicates the summary of the input–output relationship between the stimulation intensity (x-axis) and cEPSC amplitude (y-axis) of 175 neurons from 16 capsaicin-treated and 6 vehicle-treated rats with SNL and 67 neurons from 4 capsaicin-treated and 4 vehicle-treated rats without SNL (non-SNL). Whereas the mean cEPSC amplitude of the right CeC neurons was markedly and significantly larger than that of the left CeC neurons in the vehicle-treated SNL rats (filled and open large triangles in Figure 3B), there was no significant difference between the cEPSC amplitudes of the right and left CeC neurons at any simulation intensity from 50 to 1000 μA in the capsaicin-treated SNL group (filled and open large circles; Figure 3B). In rats with SNL, we failed to observe any significant differences between paired-pulse ratio (PPR) values in the right and left CeC neurons from either capsaicin- or vehicle-treated rats at any of the stimulation intensities examined (50–1000 μA; Figure 3C shows the results of the stimulation at 1 mA). We also failed to detect any significant differences in the resting membrane potential and whole-cell capacitance between right and left CeC neurons from capsaicin- and vehicle-treated rats (Table 1). To examine whether neonatal capsaicin treatment itself affects LPB-CeC transmission even without SNL, 32 and 35 neurons were recorded from capsaicin- and vehicle-treated rats, respectively. We found no significant difference between left and right CeC and also between capsaicin-treated and vehicle-treated groups (Figure 3B, smaller markers). The PPR from these rats was slightly but significantly larger than rats with SNL, which was not related to capsaicin treatment and to the side of the neuron (Figure 3C). This might explain slightly and non-significantly smaller cEPSC amplitude regardless of capsaicin treatment and side of recording (Figure 3B). These results suggest that neonatal capsaicin treatment alone does not affect the left-right balance of the LPB-CeC transmission and that the absence of post-SNL potentiation in the capsaicin treated rats results from specific effect of neonatal capsaicin related to SNL-induced changes rather than its non-specific lateralized effects on the right LPB-CeC pathways.

**Table 1:** Effect of capsaicin on membrane properties of CeC neurons

		**Resting membrane**	**Input resistance**	**Whole-cell**
**Treatment**	**Recorded side**	**potential (mV)**	**(MΩ)**	**capacitance (pF)**
Capsaicin	Left (n = 84)	−58.5 ± 0.9	195.2 ± 9.2	14.5 ± 0.9
	Right (n = 116)	−59.9 ± 0.7	214.2 ± 9.1	15.3 ± 0.7
Vehicle	Left (n = 37)	−60.4 ± 1.1	228.7 ± 23.5	16.6 ± 1.4
	Right (n = 42)	−60.7 ± 1.3	245.2 ± 19.6	15.6 ± 1.3

Membrane properties and whole-cell capacitance of CeC neurons from either side in capsaicin- or vehicle-treated rats recorded 7–9 days after spinal nerve ligation. Values are expressed as the mean ± SEM and were measured immediately after establishment of the whole-cell mode. Junction potentials were not compensated. No significant differences in the resting membrane potential, input resistance or whole-cell capacitance between the treatment groups or between the left and right CeCs were observed (ANOVA followed by Bonferroni post-hoc test). Numbers in parentheses indicate the number of neurons.

### Effect of capsaicin treatment on single-fiber EPSC (sfEPSC) amplitude

**Figure 4:**
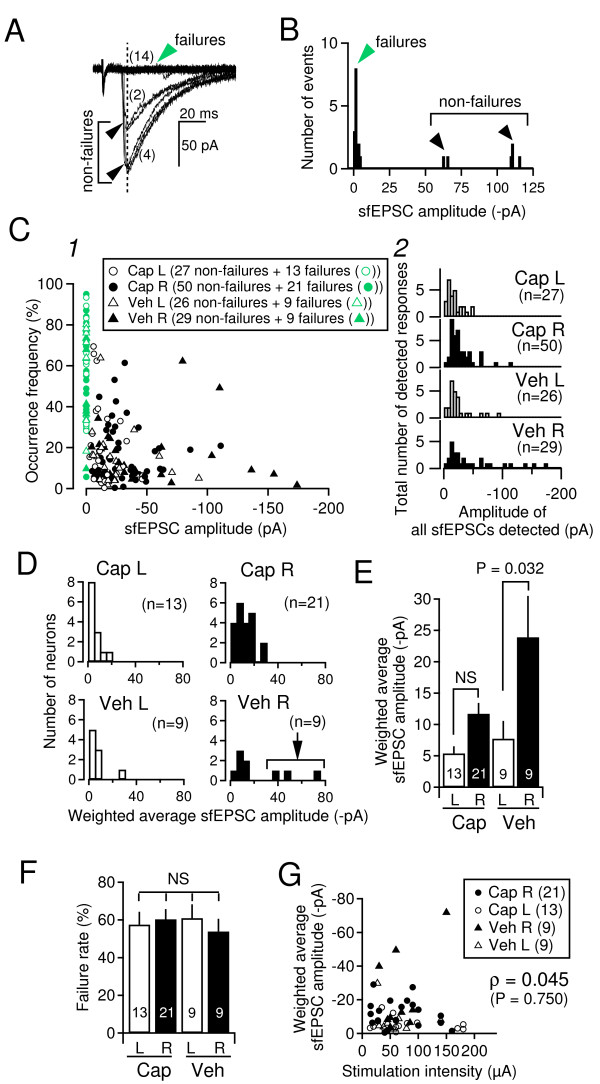
**Analyses of EPSC amplitude evoked by the “minimal stimulation” protocol.A**, an overlay of 20 traces of evoked responses to "minimal stimulation" with a theta-pipette electrode recorded in the right CeC from a capsaicin-treated rat. The stimulation intensity was fixed at 20 μA. There were "failure" (no evoked response; green filled arrowhead) and “non-failure” (black filled arrowheads) responses. Numbers in the parenthesis indicate the number of traces grouped according to the event amplitude. **B**, amplitude distribution histogram of sfEPSCs recorded from the neuron shown in A based on the responses to 20 consecutive stimulation trials (traces contaminated with spontaneous EPSCs or irregular asynchronous noisy events were carefully excluded by visual observation). The peaks in the histogram were classified into "failure" and “non-failure” sfEPSC groups. In this neuron and in many other neurons, “non-failure” sfEPSCs were composed of sfEPSCs with distinct amplitudes (the peaks around distinct values). The estimated "area" of these peaks was calculated by fitting Gaussian distributions onto each peak and the occurrence frequency of the responses belonging to each distinct peak was calculated by dividing each area by the summation of the areas of all peaks identified. In the case of this neuron in B, the occurrence frequency of the failure responses was 69.8%, that of the smaller peak was 9.2% and that of the larger peak was 20.9%. **C**, distribution of the sfEPSC amplitude for 4 groups. C1, relationship between sfEPSC amplitude of each identified response (x-axis) and its occurrence frequency (y-axis). All identified responses in a total of 132 neurons belonging to different groups are plotted. C2, histograms for the all detected peaks of each sfEPSC amplitude for distinct 4 groups. Y-axis, the pooled total number of detected responses. "Cap L" and "Cap R", neurons recorded in the left (open bars) and right (filled bars) CeC, respectively, from capsaicin-treated SNL rats. "Veh L" and "Veh R", neurons recorded in the left (open bars) and right (filled bars) CeC, respectively, from vehicle-treated SNL rats. The numbers in parenthesis indicate the number of identified responses; in most of the cases, multiple responses were identified from single neurons. **D**, histograms showing the distribution of weighted average sfEPSC amplitude for each neuron recorded from left (L, open bars) and right (R, filled bars) CeC from capsaicin (Cap)- and vehicle (Veh)-treated rats. Y-axis indicates the number of neurons. The numbers in parenthesis indicates the number of neurons. **E**, summary of the weighted average sfEPSC amplitude from the neurons recorded from left (L, open bars) and right (R, filled bars) CeC from capsaicin (Cap)- and vehicle (Veh)-treated SNL rats. Mean ± SEM. The numbers in the bars show the number of neurons. ANOVA followed by Bonferroni correction. **F**, Summary of the failure rate for each neuron group. The abbreviations and the numbers are the same as in E. Mean ± SEM. ANOVA followed by Bonferroni correction. **G**, Relationship between intensity of the minimal stimulation (x-axis) and weighted average sfEPSC amplitude of each neuron. The abbreviations and the numbers are the same as in E. Spearman's rank correlation coefficient (ρ) was 0.045, which was not significant.

The results described above indicate that neonatal capsaicin-treatment prevents establishment of the synaptic potentiation induced by unilateral SNL. Our previous study and the present results indicate that the synaptic potentiation in the SNL model primarily results from increased postsynaptic responses, without detectable changes in the PPR (see Figure 3C in [15]). To elucidate the mechanisms underlying the lack of LPB-CeC synaptic potentiation in the capsaicin-treated SNL rats, we analyzed the responses to the single-fiber minimal stimulation protocol in the SNL rats with and without capsaicin-treatment. For this purpose, we used theta pipette electrodes because they enabled passing currents within a highly limited area (~1 μm in diameter), thus activating a very limited number (one or a few) of afferent fibers [25]. Figure 4A shows superimposed responses of a right CeC neuron in capsaicin-treated SNL rats to consecutively delivered theta-pipette electrode stimulation. The intensity was carefully fixed at a level slightly above the level giving "all-failure" responses (minimal intensity). As it is highly likely that the non-failure events with constantly small amplitude resulted from activation of a single afferent fiber, we termed these responses as "single-fiber EPSC" (sfEPSC). The sfEPSC waveforms consisted of failure responses (green arrowhead in Figure 4A) and non-failure responses (filled arrowhead in Figure 4A). Of these non-failure responses, events with discrete amplitudes that could be classified into distinct groups were also observed in many neurons, even in response to the stimulation at a fixed minimal intensity (e.g., two different types of events with different amplitudes and different occurrence frequencies were observed among non-failure responses in Figure 4A). Figure 4B shows the amplitude distribution of the sfEPSC waveforms depicted in Figure 4A. In addition to the peak for the "failure" events (green arrowhead at approximately 0 pA), two distinct peaks for non-failure events were observed (one approximately 60 pA, another approximately 110 pA).

Based on such analysis of events evoked by the minimal stimulation protocol, we analyzed the amplitude and occurrence frequency of such non-failure events as well as the frequency of failure responses during 7–25 (18.0 ± 0.6; 14–25 for 92% of cases) repeated trials in a total of 34 neurons from the right and left CeC (of which neurons, cEPSC was simultaneously recorded in 7 cells described above) from 23 capsaicin-treated rats and 18 neurons from the right and left CeC (of which neurons, cEPSC was simultaneously recorded in 6 cells) from 13 vehicle-treated rats that underwent the SNL. Figure 4C1 displays the mean sfEPSC amplitude (x-axis) and its occurrence frequency (y-axis) for all detected responses from all neurons analyzed. The amplitude of sfEPSC was estimated by fitting a Gaussian peak onto the histogram such as that in Figure 4B. Figure 4C2 indicates the distribution of these all identified responses plotted in Figure 4C1. These plots in Figure 4C1 and 2 indicate that large-amplitude events could be mostly (but not exclusively) found in the right CeC in both capsaicin- and vehicle-treated SNL animals. In particular, though the occurrence frequency was small, a part of right CeC neurons showed extremely large sfEPSC (Figure 4C1, filled triangles; Figure 4C2, the histogram "VehR").

The contribution of these single-fiber transmissions to the whole synaptic transmission could be determined by considering both the amplitude and its occurrence frequency. A large sfEPSC would not affect so much the cEPSC amplitude if its occurrence frequency is low. Therefore, to estimate "expected value" of the sfEPSC amplitude in each neuron, we calculated "weighted average" of sfEPSC amplitude by summing the product of sfEPSC amplitude of each response and its occurrence frequency for all distinct-amplitude responses detected in each neuron. This "weighted average sfEPSC amplitude" shows the expected value of amplitude of the response of each neuron by taking the amplitude and its probability into consideration. Figure 4D indicates the distribution of this weighted average sfEPSC amplitude for the neurons from four groups. Only in the neurons in right CeC from vehicle-treated rats showed large weighted average amplitude (arrow in "Veh R" in Figure 4D). Indeed, the weighted average of sfEPSC amplitude of the right CeC neurons from vehicle-treated rats was significantly larger than that of the left CeC neurons from the same group (Figure 4E). In contrast, we failed to detect significant difference in weighted average of sfEPSC amplitude between right and left CeC in the neurons from capsaicin-treated rats (Figure 4E). There was no significant difference in the failure rate between all four groups (Figure 4F), suggesting that the release probability of the afferent fibers is not essentially affected by SNL and capsaicin treatment, a finding consistent with the lack of significant difference in the PPR between right and left CeC from capsaicin- and vehicle-treated animals estimated on the basis of cEPSC amplitude (Figure 3C). Such differences in the sfEPSC amplitude between the groups were unlikely to have resulted from the differences in the stimulation intensity used because there was no correlation (Spearman's rho = 0.045; P = 0.75) between the weighted average sfEPSC amplitude for each neuron and the stimulation intensity used for each recording (Figure 4G). These results of cEPSC and weighted average sfEPSC amplitude in the right CeC of capsaicin- and vehicle-treated rats suggest that the amplitude of cEPSC is a linear and ergordic summation of the sfEPSC amplitude in this system and that the effect of neonatal capsaicin treatment in preventing the SNL-induced potentiation of LPB-CeC transmission could be attributed to its influence on single fiber transmission levels.

## Discussion

Neonatal capsaicin-treatment in rats resulted in an almost complete absence of sensitivity to acute capsaicin application and nearly unaffected mechanical allodynia after SNL. These observations are in a good accordance with previous studies showing that the expression of mechanical allodynia does not require nociceptive C-fiber afferents [26-28]. The novel finding of this study is that the synaptic potentiation of the LPB-CeC excitatory synapses that is shown to occur after one week of mechanical allodynia following SNL [15] failed to occur in the rats with neonatal capsaicin-treatment, despite a clear manifestation of mechanical allodynia. This result strongly points to a promoting role of C-fiber-mediated inputs in the establishment of pain-induced plasticity in the amygdala.

### Limited role of TRPV1-expressing C-fiber afferents in the establishment of mechanical allodynia after SNL

These results indicate a lack of influence of neonatal capsaicin-treatment on the expression of withdrawal spinal reflexes both before and after SNL. It is not likely that the capsaicin treatment was insufficient in abolishing the nerves expressing TRPV1 because the eye-wiping behavior in response to capsaicin instillation disappeared almost completely in the capsaicin-treated rats. The conclusions drawn from these observations are three-fold. First, the C-fiber afferents expressing TRPV1 channels are not necessary for the withdrawal reflex to strong mechanical stimulation, which are observed before the SNL and in the uninjured side after SNL. This result supports the common view of a major involvement of Aδ-fiber-mediated nociceptive high-threshold mechanoreceptor afferents in this reflex in non-neuropathic rats and in the uninjured hindlimb [29]. Second, the inputs carried by the C-fiber afferents are not essential for the presumed reorganization of the spinal cord local network underlying the development of tactile allodynia and other hyperalgesic behaviors after SNL [17,24]. These two conclusions are in line with previously published reports indicating limited involvement of C-fiber afferents in the establishment of tactile allodynia [17,24,30,31]. Third, C-fiber afferents are not necessary for the aberrant light touch-induced withdrawal reflex observed in animals after SNL, in line with a common view that Aβ-fibers carrying information of innocuous touch are involved in such tactile allodynia. This conclusion is in good accordance with the absence of the effect of resiniferatoxin, a TRPV1 agonist almost equipotent to capsaicin but with a higher potency in inducing its long-lasting desensitization [32], on tactile allodynia in SNL model in adult rats [17], and with the unaffected robust mechanical allodynia both before and after partial sciatic nerve ligation in mice genetically lacking TRPV1 channel proteins [33]. Altogether, the present results confirm that the role of TRPV1-expressing C-fiber afferents in the chronic pain-induced facilitation of nociceptive behaviors seems to be highly limited.

### How could C-fiber-mediated inputs affect LPB-CeC synaptic potentiation?

Recent anatomical studies indicate that a large portion (95%) of NK1 receptor-positive projecting neurons in the lamina I that receive C-fiber afferents [34,35] project to the LPB [36], but only a small portion to the thalamus [5]. These observations suggest that the superficial dorsal horn neurons receiving C-fiber-mediated inputs are the major source of nociception-related inputs to the LPB. However, as discussed above, nerve injury in the SNL model induces reorganization of the spinal cord local circuit, allowing Aβ-fiber-mediated low-threshold inputs to elicit aberrant hyperalgesic spinal reflexes [37,38]. The question here is why did the lack of inputs mediated by TRPV1-expressing C-fibers, which had no influence on allodynia, have an inhibitory influence on the processes involved in synaptic potentiation in the LPB-CeC synapse in the animals with neuropathic pain?

The first possibility is that the activity of C-fiber afferents is also increased in SNL rats, despite a lack of influence on tactile allodynia and that the capsaicin-treatment eliminated this influence, preventing LPB-CeC potentiation. L5 spinal nerve ligation results in Wallerian degeneration of A-fibers in the L5 but is also accompanied by an increased spontaneous activity in the C-fiber afferents in the adjacent L4 [39]. This suggests that injured A-fibers can stimulate nearby C-fibers after nerve injury, presumably through releasing substances following degeneration. As such, it is expected that the increased spontaneous activity of adjacent C-fibers in the SNL animals would also increase the activity of LPB-projecting lamina I neurons, and the lack of such C-fibers by capsaicin treatment is, therefore, likely to result in lesser activation of these neurons. In addition, spared afferent neurons, such as those in the L4 DRG in the L5-nerve injured model, show activation of inflammation-related molecules, suggesting that inflammatory responses in the non-injured neurons might play core roles in the nerve injury-induced hyperalgesic symptoms [40]. It is, therefore, possible that denervation of C-fiber afferents might reduce such inflammatory responses in the spared neurons, hence resulting in less potentiation of LPB-CeC transmission following SNL.

The second possibility is that the aberrant sprouting-based reorganization of the dorsal horn network [37,38] would also influence the activity of the spino-parabrachial ascending neurons. Keller et al. [41] recorded discharges of lamina I neurons antidromically excited by LPB stimulation in the SNL model and found that these neurons showed increased responses to touch stimulation and showed spontaneous burst discharges. Though they did not examine the type of primary afferent fibers that these neurons receive, they attributed this increase to spinal network reorganization allowing Aβ afferents to directly and aberrantly excite lamina I neurons projecting to the LPB [41]. Though it remains to be an open question whether neonatal capsaicin treatment prevents such an increase in the ascending activity in SNL animals, such attenuated activity of LPB-projecting fibers, if it occurs, would prevent synaptic potentiation in the SNL models.

The third possibility stems from the recently acknowledged fact that the TRPV1 channels are also expressed and functional in the brain of adult and neonate animals [42-46]. For example, activation of TRPV1 channels with capsaicin modulates synaptic plasticity in the lateral amygdala [47]. Although it remains undetermined whether neonatal capsaicin treatment also inactivates the TRPV1-expressing neurons within the brain in a similar manner to those in the periphery, it is probable that peripherally administered capsaicin might pass through the brain blood barrier (BBB) because 1) the BBB is more permeable in the neonates due to poorly developed astrocyte-vessel contacts, and 2) it has recently been shown that sole activation of TRPV1-expressing C-fibers or peripheral nerve injury itself is sufficient to cause long-lasting dysfunction of the BBB [48]. If such irreversible inactivation of TRPV1 channels in brain networks occurs in the neonatal capsaicin-treated animals, it is possible that the synaptic organization and excitability of the amygdala neurons are directly or indirectly affected. However, as the neonatal capsaicin-treatment without postnatal SNL did not produce any imbalance between the right and left LPB-CeC transmissions, it is unlikely that central denervation of TRPV1-expressing neurons is the direct cause of the absence of hemilateral potentiation after SNL in capsaicin-treated group.

Interestingly, the analysis of single-fiber EPSC amplitude indicated that the absence of synaptic potentiation in the capsaicin-treated rats is characterized with smaller number of large-amplitude sfEPSCs and also with their lower occurrence frequency than in the vehicle-treated rats, both of which resulted in significantly smaller weighted average sfEPSC amplitude in the right CeC in capsaicin-treated SNL rats. This observation suggests that the establishment of synaptic potentiation in SNL models requires both an increased single-fiber responses in a subset of afferents and also an increase in the occurrence probability of the synaptic transmission between such fibers and CeC neurons. It is likely that the elimination of C-fiber afferents to the spinal dorsal horn prevented such changes. The synaptic mechanism enabling such potentiation awaits further investigation in the future.

### Functional significance of this finding

The mechanism and functional significance of central integration of distinct but mutually related nociceptive signals of distinct peripheral receptor origins in higher brain centers remain yet to be determined. For example, it is not well understood how the information originating from nociception-specific neurons and wide-dynamic range neurons is integrated in the CNS. In general, the central perception and recognition of pain depend on the simultaneous activation of a widely distributed "pain matrix" that is independently but coordinately activated by divergent nociceptive inputs [49]. The results of this study indicate that central sensitization of the network underlying nociception-induced emotion can be dissociated from the allodynic responses at the spinal cord after selective denervation of a specific type of afferent fiber. This conclusion implies a novel idea that there could be a subset of nociceptive fibers specifically linked to the generation of pain-induced negative emotion, of which the amygdala network is the principal target. In this case, the C-fiber afferents or their subsets seem to play this role. This idea is not without precedent; in other sensory systems such as the olfactory and gustatory systems, there is a distinction at the primary sensory levels in the sensory cells and afferent projections between aversive and palatable information sent to specific brain regions handling such information of distinct values [1,50]. The present results indicate that, in the nociceptive system, the TRPV1-expressing C-fibers would play predominant role in the long-lasting potentiation of the nociception-emotion link [13]. Pharmacological manipulation of such a system would be of potent therapeutic value, as it would allow selective attenuation of the affective and suffering aspect of pain while leaving the sensorial function of detecting noxious events intact.

## Conclusions

TRPV1-expressing C-fibers play a promoting and essential role in amygdala synaptic potentiation and its consolidation of the transmission between input arising from the LPB and CeC neurons in the SNL models. This conclusion suggests a specific role of C-fiber afferents in the establishment of nerve injury-driven potentiation of the nociception-emotion link.

## Methods

### Postnatal capsaicin treatment

All animal manipulation was approved by the Animal Care Committee of the Jikei University School of Medicine and conformed to the Guiding Principles for the Care and Use of Animals in the Field of Physiological Sciences of the Physiological Society of Japan (1998) and to the guidelines of the International Association for the Study of Pain [51].

Neonatal Wistar rats of either sex were anesthetized with diethyl ether 12–48 hours after birth and either a capsaicin solution (capsaicin-treated group) or vehicle solution (vehicle-treated group) was subcutaneously injected. The capsaicin injection solution contained 1% or 5% capsaicin (Sigma), 10% ethanol and 10% Tween-80 in saline. The vehicle solution contained 10% ethanol and 10% Tween-80 in saline. The injection volume was adjusted in each animal according to its body weight and solution concentration so that the dose of capsaicin in this solution became 50 mg/kg in the same manner as a previous report showing maximal degeneration of unmyelinated afferent neurons [52]. The pups were placed on a soft water bed heated at approximately 37°C until recovery from anesthesia and were returned to their mothers afterwards. They were weaned approximately at 3 weeks and maintained under standard laboratory conditions under a 12 hr light/dark cycle. They had free access to food and drinking water. There were no crucial differences in general behavior or food intake between these two groups. The body weights at the third postnatal week were 29.3 ± 0.7 g (mean ± standard error of the mean; n = 27) and 26.7 ± 1.3 g (n = 13), for the capsaicin- and vehicle-treated groups, respectively. Because we could not detect any apparent difference between the results from male and female rats, their data were pooled. All animals of both capsaicin- and vehicle-treated groups survived until the SNL and decapitation for electrophysiological recordings (see below).

### Evaluation of capsaicin-induced eye-wiping

The sensitivity to capsaicin in each animal belonging to either the capsaicin- or vehicle-treated group was evaluated by observing the behavioral responses to instillation of capsaicin solution to the cornea according to a previously described method ("eye-wiping test" in [53,54]). The 0.01% capsaicin eye instillation solution was made by diluting the stock solution containing 1% capsaicin, 10% ethanol and 10% Tween-80 with saline to 1:100. The solution of the same content but without capsaicin was called "solvent" and was used as a control for capsaicin application. On postnatal day 19–20, an approximately 10 μl drop of 0.01% capsaicin solution or of solvent was applied to the eyes of an unrestrained rat. The interval between instillations in one rat was more than 30 min. The order of application of capsaicin solution and solvent, as well as that of right and left eyes, was randomized in a blind manner by two experimenters. The number of eye-wiping behaviors in the first 2 min after the application was counted by visual inspection of one of these experimenters also in a blind manner.

### Neuropathic pain model

At postnatal day 20–28, rats were separated from their dam and anesthetized initially with diethyl ether and then mounted on a surgical platform in a prone position with the limbs fixed while being kept continuously anesthetized with isoflurane inhalation (1.5-2% in 100% O_2_). A longitudinal incision (0.5-1 cm in length) was made at the midline of the lower lumbar using a sterile surgical blade. The left paraspinal muscles were isolated and the left L6 transverse process was exposed. Under a dissecting microscope, the left L6 transverse process covering the L4 and L5 spinal nerves was carefully removed. The left L5 spinal nerve was isolated and tightly ligated with 6–0 silk threads. After surgery, the muscles were sutured in layers, the skin was closed using silk thread (4–0), and anesthesia was discontinued. Animals fully recovered from anesthesia within 30 min and showed no signs of distress as evidenced by the monitoring of breathing patterns, grooming behavior and locomotion in the cage. The rats were housed in cages filled with soft cushion-like flooring in a temperature- (approximately 25°C) and humidity- (approximately 50%) controlled room until decapitation as described below. Water and food were available ad libitum, and there was no apparent difference in daily consumption of water and food and in body weight measured before decapitation between the groups. Behavior was monitored regularly, and no aberrant symptoms arising from excessive nociception were observed except for a mild deformity of the lesioned paw [16].

### Von Frey filament test

The paw withdrawal threshold in response to mechanical stimuli was evaluated by well-trained experimenters, according to the previously reported method [15]. Mechanical stimuli were applied using von Frey filaments of different rigidity (0.4 - 15.0 g). Each rat was placed on a metal mesh floor (25 cm x 25 cm) and a von Frey filament was applied manually from beneath. The 50% threshold for the paw withdrawal behavior (50%-paw withdrawal threshold, PWT) was estimated by the up-and-down method [55]. Care was taken to reduce the number of trials to avoid unnecessary pain sensations. The tests were performed 3 times for each rat; immediately before the SNL, once within post-operational day 2–4, and once immediately before decapitation for the slice preparation.

### Preparation of transverse brain slices

Immediately after the final von Frey filament test on post-operational day 7–9, the rats were decapitated under isoflurane anesthesia (5% in 100% O_2_). We obtained coronal brain slices both ipsilateral (left) and contralateral (right) to the operation side (left) from the capsaicin-treated group (56 unilateral slices from 27 rats) and from the vehicle-treated group (31 unilateral slices from 13 rats) according to previously described procedures [15]. Briefly, a transverse block of forebrain containing the amygdaloid complex was dissected out and cut at the midline. The dissected hemisphere was secured on the cutting stage of a vibrating blade slicer (DSK-1000, Dosaka EM) with the rostral end upwards. Coronal slices of 400-μm thickness containing the amygdala were cut in ice-cold cutting artificial cerebrospinal fluid (ACSF) composed of (in mM) 125 NaCl, 3 KCl, 0.1 CaCl_2_, 5 MgCl_2_, 1.25 NaH_2_PO_4_, 10 D-glucose, 0.4 L-ascorbic acid and 25 NaHCO_3_ (pH 7.4 bubbled with 95% O_2_ + 5% CO_2_; osmolarity, approximately 310 mOsm/kg). The slices were first incubated in a holding chamber with a constant flow of standard ACSF, of which the concentrations of CaCl_2_ and MgCl_2_ were 2 mM and 1.3 mM, respectively, at 37°C for 30 to 45 min. The slices were kept at room temperature (20-25°C) in the same chamber until the electrophysiological recording. Each slice was transferred to a recording chamber (approximately 0.4 ml volume) and fixed with nylon grids to a platinum frame. The slice was submerged in and continuously superfused at a rate of 1–2 ml/min with standard ACSF. To isolate excitatory synaptic inputs, 100 μM picrotoxin (Sigma) and 1 μM strychnine HCl (Sigma) were dissolved in ACSF and bath-applied throughout the recording.

### Patch-clamp recordings

Neurons in the CeC were visually identified under an upright microscope (BX-51WI, Olympus) with oblique illumination or infrared interference contrast (IR-DIC) optics. Images were captured using a CCD camera (IR-1000, DAGE-MTI) and stored digitally on a computer. Whole-cell transmembrane current was recorded from neurons in the left and right CeC (i.e., ipsi- and contralateral, respectively, to the SNL). Patch-clamp electrodes were made from borosilicate glass pipettes (1B120F-4; World Precision Instruments). The composition of the internal solution was (in mM) 120 potassium gluconate, 6 NaCl, 1 CaCl_2_, 2 MgCl_2_, 2 ATP Mg, 0.5 GTP Na, 12 phosphocreatine Na_2_, 5 EGTA, 5 QX-314 and 10 HEPES hemisodium (pH 7.2 as adjusted with KOH; osmolarity, approximately 310 mOsm/kg) was added to the internal solution. The tip resistance of the electrode was 3–6 MΩ. The evoked cEPSCs were recorded at a holding potential of −70 mV. The input resistance, resting membrane potential and whole-cell capacitance were measured immediately after the establishment of whole-cell mode by membrane rupture. Membrane currents were recorded using an Axopatch 200B amplifier (Axon Instruments), low-pass filtered at 2 kHz and digitized at 4 or 10 kHz and 16-bit resolution with a PowerLab interface (ADInstruments).

All recordings were made at room temperature (20-25°C). The order of recordings from the right and left amygdala was randomized to avoid side-dependent differences due to changes in the viability of neurons during the time from slice preparation to recording. All compounds except those noted above were purchased from WAKO (Osaka, Japan) or Nacalai Tesque (Kyoto, Japan).

### Afferent pathway stimulation

To activate action potential-dependent glutamate release from afferent fibers arising from the LPB, we carefully located the stimulating electrode on the fiber tract ventromedial to the CeC under microscopic control. The following two types of stimulation protocol with different electrodes were performed. (1) Gross stimulation to evoke cEPSCs. A bipolar concentric steel electrode (interpolar distance, approximately 100 μm; Unique Medical, Tokyo, Japan) was used, and the stimulation intensity was set at 50, 100, 200, 400, 800 and 1000 μA. The pulse duration was 100 μs. The cEPSC amplitude increased in a graded manner as the stimulation intensity was elevated, suggesting that the amplitude depended on the number of afferent fibers recruited in response to a single stimulation. Double pulses with an inter-stimulus interval of 50 ms were delivered to calculate the PPR of the cEPSC amplitude by normalizing the amplitude of second cEPSC by that of first cEPSC [15]. (2) Minimal stimulation to evoke sfEPSC [56]. The stimulation electrodes for minimal stimulation were made from "theta" glass pipettes (TST150-6, World Precision Instruments), which were pulled with a similar procedure to that used for making patch pipettes. The theta stimulation pipette had a very small interpolar distance at the tip (approximately 1 μm) and was filled with ACSF. The stimulation intensity was first set at a moderate value and gradually decreased while the evoked EPSCs were observed. Then, the sfEPSC was identified in an "all-or-none" manner by the appearance of EPSCs with a similar waveform and latency at a consistent probability in response to a fixed stimulation intensity [25].

### Data and statistical analysis

The recorded membrane current was analyzed off-line with Igor Pro 5 (WaveMetrics, OR, USA) using procedures written by F. K. The peak amplitude was measured based on the averaged waveform of 8 consecutive evoked cEPSCs. Values are expressed as the mean values ± standard error of the mean (SEM). Differences in the values were compared using one-way analysis of variance (ANOVA) followed by Bonferroni post-hoc test and Mann–Whitney *U* test. Differences with a probability (*P*) less than 0.05 were considered significant.

## Abbreviations

CeC, Capsular part of the central nucleus of the amygdala; Cap, Capsaicin; Veh, Vehicle; LPB, Lateral parabrachial nucleus; cEPSC, compound excitatory postsynaptic current; sfEPSC, single-fiber excitatory postsynaptic current; SNL, Spinal-nerve ligation (or spinal-nerve ligated).

## Competing interests

The authors declare no conflict of interest for the materials and techniques used in this study.

## Authors’ contributions

AN, YT and MN carried out all experiments and data analyses. AN, YT and FK wrote the text, RI and FK designed the study. All 5 authors participated in the discussion. All authors read and approved the final manuscript.
